# Predictors of Early and Late Infarct Growth in DEFUSE 3

**DOI:** 10.3389/fneur.2021.699153

**Published:** 2021-07-01

**Authors:** William J. Tate, Laura C. Polding, Søren Christensen, Michael Mlynash, Stephanie Kemp, Jeremy J. Heit, Michael P. Marks, Gregory W. Albers, Maarten G. Lansberg

**Affiliations:** ^1^Stanford University School of Medicine, Stanford, CA, United States; ^2^Stanford Stroke Center, Palo Alto, CA, United States; ^3^Department of Radiology, Stanford University School of Medicine, Stanford, CA, United States

**Keywords:** stroke, infarct growth, reperfused, collaterals, brain imaging (CT and MRI)

## Abstract

**Introduction:** The goal of this study is to explore the impact of reperfusion and collateral status on infarct growth in the early and late time windows.

**Materials and Methods:** Seventy patients from the DEFUSE 3 trial (Endovascular Therapy Following Imaging Evaluation for Ischemic Stroke) with baseline, 24-h, and late follow-up scans were evaluated. Scans were taken with DWI or CTP at time of enrollment (Baseline), with DWI or CT 24-h after enrollment (24-h), and with DWI or CT 5 days after enrollment (Late). Early infarct growth (between baseline and 24-h scans) and late infarct growth (between 24-h and late scans) was assessed for each patient. The impact of collateral and reperfusion status on infarct growth was assessed in univariate and multivariate regression.

**Results:** The median early infarct growth was 30.3 ml (IQR 16.4–74.5) and the median late infarct growth was 6.7 ml (IQR −3.5–21.6) in the overall sample. Patients with poor collaterals showed greater early infarct growth (Median 58.5 ml; IQR 18.6–125.6) compared to patients with good collaterals (Median 28.4 ml; IQR 15.8–49.3, unadjusted *p* = 0.04, adjusted *p* = 0.06) but showed no difference in late infarct growth. In contrast, patients who reperfused showed no reduction in early infarct growth but showed reduced late infarct growth (Median 1.9 ml; IQR −6.1–8.5) compared to patients without reperfusion (Median 11.2 ml; IQR −1.1–27.2, unadjusted *p* < 0.01, adjusted *p* = 0.04).

**Discussion:** In the DEFUSE 3 population, poor collaterals predict early infarct growth and absence of reperfusion predicts late infarct growth. These results highlight the need for timely reperfusion therapy, particularly in patients with poor collaterals and indicate that the 24-h timepoint is too early to assess the full impact of reperfusion therapy on infarct growth.

**Clinical Trial Registration:** http://www.clinicaltrials.gov, Unique identifier [NCT02586415].

## Introduction

In the DEFUSE 3 (Endovascular Therapy Following Imaging Evaluation for Ischemic Stroke) trial, endovascular therapy had a very large beneficial effect on long-term functional outcomes. Surprisingly, endovascular treatment did not have an effect on infarct growth. This might be because the follow-up MRI, used to assess infarct growth, was obtained relatively early (24 h) after randomization (1). In this study we address this limitation by analyzing infarct growth in the subset of patients who underwent an additional scan for clinical purposes beyond the 24-h time point.

Two factors that likely influence infarct growth are reperfusion and collateral blood flow. Reperfusion is a strong predictor of clinical outcome and may influence infarct growth over an extended time period (2, 3). Collateral blood flow is dynamic and can change over time, influencing its impact on infarct growth (4). The hypoperfusion intensity ratio (HIR), derived from CT perfusion (CTP) or MR perfusion imaging, is a measure of collateral status that is associated with infarct growth and with the persistence of a favorable diffusion weighted imaging (DWI)/perfusion weighted imaging (PWI) mismatch profile (5, 6).

The goal of this study is to explore the impact of reperfusion and collateral status on infarct growth in the early (between baseline and 24 h) and late (between 24 h and 5 days) time windows. Our hypothesis is that collateral status is a stronger predictor of early infarct growth while reperfusion status is a stronger predictor of late infarct growth.

## Materials and Methods

The data that support the findings of this study are available from the corresponding author on reasonable request.

### Patient Demographics

The inclusion criteria, study design, and primary results of the DEFUSE 3 trial have been reported previously (1). We identified all cases in DEFUSE 3 with an unscheduled late scan, defined as either CT or MR imaging obtained after the 24-h follow-up scan but within 2 weeks of stroke onset. For cases with multiple late scans, we included only the scan closest to day 5. We excluded cases with parenchymal hematoma because the ischemic infarct volume cannot be accurately measured in this setting. Only patients with a baseline, a 24-h, and a late scan were included in this study.

### Image Analysis and Definitions

The DEFUSE 3 imaging protocol has been previously reported (1). Infarct volumes were outlined manually on CT and MRI using OsiriX software. Early infarct growth was defined as the change in infarct volume between the infarct core assessed with DWI or CTP on the baseline scan and the infarct volume assessed with DWI or CT on the first follow-up scan, obtained 24 h after randomization. Late infarct growth was defined as the change in infarct volume between the 24-h scan, and the DWI or CT late unscheduled scan. Baseline collateral status was measured using HIR as assessed on baseline CTP and MR perfusion studies and was defined as the proportion of the Tmax >6 s lesion with a Tmax delay of >10 s (5). An HIR ≤0.4 was categorized as good baseline collateral status and HIR >0.4 categorized as poor baseline collateral status. This binary threshold for HIR was an optimal predictor of collateral status based on digital subtraction angiography (7). Reperfusion was defined as >90% reduction in the volume of tissue with perfusion delay (Tmax > 6 s) between baseline and 24 h, or complete recanalization on the 24-h CT or MR angiogram.

### Statistical Analyses

We compared demographic, clinical, and neuroimaging variables using chi-square and Wilcoxon rank-sum tests. The associations between the following candidate predictor variables and early infarct growth were tested in univariate analysis: age, baseline NIHSS score, glucose, time from stroke onset to baseline imaging, time from stroke onset to 24-h imaging, baseline HIR, reperfusion status, and baseline infarct volume. The associations between these same candidate predictor variables and late infarct growth were tested in univariate analysis with the replacement of baseline NIHSS score with 24-h NIHSS score, time from stroke onset to 24-h imaging replaced with time from 24-h scan to late scan, and baseline infarct volume replaced with 24-h infarct. Treatment arm was not assessed as a predictor variable as it strongly correlates with reperfusion status because the majority of patients in the endovascular therapy arm had successful reperfusion. Similarly, gender was not assessed as a predictor variable as it has previously been shown to correlate strongly with HIR in the DEFUSE 3 data (8). For both early and late infarct growth, variables significant at α < 0.1 in univariate analyses were entered into a multivariate linear regression model and were retained in the model using a backwards-elimination method if they remained significant at α < 0.05. Reperfusion was included in both models because it has a known association with infarct growth (9). We also included an interaction term (reperfusion x baseline collateral status) in the models to test the hypothesis that infarct growth may be more significant in non-reperfused patients with good baseline collaterals. We defined an alpha value of <0.05 as statistically significant and report two-sided results. Alpha values between 0.05 and 0.1 were interpreted as trends of association. Statistical analysis was done using SAS 9.4 (SAS Institute Inc, Cary, NC).

## Results

Of the 182 patients enrolled in DEFUSE 3, 70 (38%) had a late scan without a parenchymal hematoma and were included in this study. Three cases were excluded due to missing reperfusion data and one was excluded due to poor image quality of the late scan, leaving 66 cases available for full analysis. The late scan imaging modality was CT for 58 patients (83%) and MRI for 12 (17%) patients. The median time from 24-h scan to that of the late scan was 72 h (IQR 52–115). Table 1 compares the demographic and imaging characteristics of all subjects who were included in this study to the remaining DEFUSE 3 subjects. Patients that were included were more likely in the medical treatment group (*p* = 0.01), did not reperfuse (*p* = 0.01), had worse NIHSS scores at 24 h (*p* = 0.04), and had a higher mortality rate at day 90 (*p* = 0.05). Treatment arm was equally distributed amongst the groups with good vs. poor collaterals with endovascular therapy in 16 (40%) of patients with good collaterals and 11 (37%) of patients with poor collaterals. Treatment arm was not equally distributed amongst the reperfusion and non-reperfusion groups with endovascular therapy in 20 (80%) of the patients with reperfusion and 8 (19%) of the patients who did not reperfuse. As previously mentioned, treatment arm was not included as a variable in the analysis.

**Table 1:** Demographic and imaging characteristics of population in this study compared to DEFUSE 3.

	**Late scans (*n* = 70)**	**Rest of DEFUSE 3 (*n* = 112)**	***p*-value**
**Demographic characteristics**			
Age, median (IQR)–yrs	71 (58–80)	70 (60–79)	0.90
Female sex, no. (%)	36 (51%)	56 (50%)	0.85
Glucose, median (IQR)	122 (108–163)	125 (109–151)	0.75
**Baseline characteristics**			
NIHSS score at baseline, median (IQR)	17 (13–20)	16 (11–21)	0.37
Imaging modality at baseline			
*CT*	55 (79%)	78 (70%)	0.19
*MRI*	15 (21%)	34 (30%)	
Time - stroke onset to baseline imaging, median (IQR)–hrs	10 (9–12)	10 (8–12)	0.96
Treatment–medical therapy no. (%)	43 (61%)	47 (42%)	0.01
Reperfusion status–Reperfused no. (%)	25 (36%)	60 (58%)	0.01
Collateral status–Good no. (%)	40 (57%)	63 (57%)	0.96
Hypoperfusion intensity ratio at baseline	0.36 (0.21–0.53)	0.37 (0.21–0.50)	0.95
Infarct volume at baseline, median (IQR) - ml	10.0 (4.6–32.9)	9.4 (2.5–23.5)	0.23
**24 h characteristics**			
NIHSS score at 24 h, median (IQR)	14 (9–20)	11 (5–19)	0.04
Imaging modality at 24 h			
*CT*	13 (19%)	17 (16%)	0.60
*MRI*	57 (81%)	92 (84%)	
Time - stroke onset to 24 h imaging, median (IQR)–hrs	36 (33–39)	37 (33–39)	0.74
Infarct volume at 24 h, median (IQR)–ml	41.2 (26.7–108.8)	36.2 (16.4–92.7)	0.12
**Late scan characteristics**			
Imaging modality of late scan			
*CT*	58 (83%)	-	-
*MRI*	12 (17%)	-	
Time from 24 h to late imaging, median (IQR)–hrs	72 (52–115)	-	-
Death at Day 90–no. (%)	19 (27%)	17 (15%)	0.05

*IQR, Interquartile range; NIHSS, National Institutes of Health Stroke Scale; CT, Computerized tomography; MRI, Magnetic resonance imaging; HIR, Hypoperfusion intensity ratio*.

**Figure 1:**
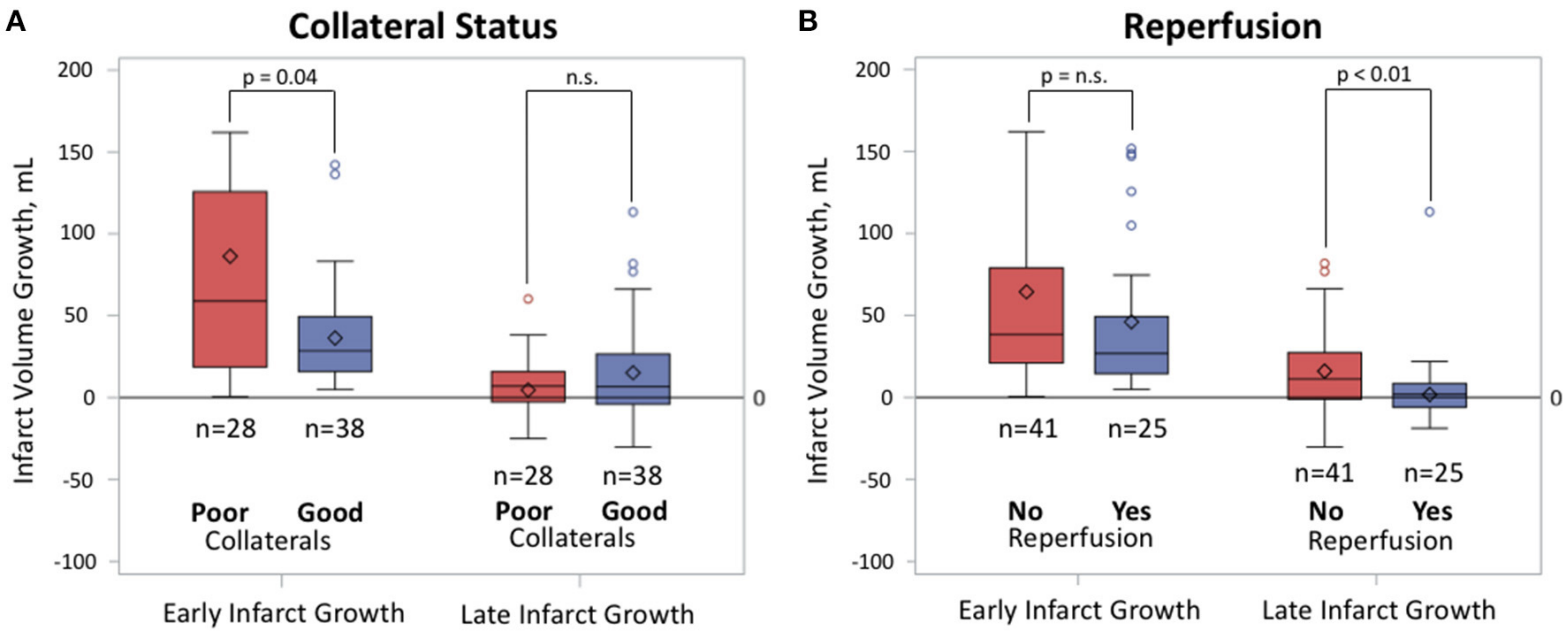
Box plot of early and late infarct growth by collateral status and reperfusion status. Black bars represent medians. Diamonds represent means. Circles represent outliers. **(A)** Good collaterals show a reduction in early infarct growth only. **(B)** Reperfusion shows a reduction in late infarct growth only.

For the 66 patients included in the full analysis, the median early infarct growth was 30.3 ml (IQR 16.4–74.5) and the median late infarct growth was 6.7 ml (IQR −3.5–21.6). Patients with poor collaterals showed greater median early infarct growth (58.5 ml; IQR 18.6–125.6) compared to patients with good collaterals (28.4 ml; IQR 15.8–49.3; *p* = 0.04, Figure 1A), but showed no difference in late infarct growth (7.0 ml; IQR −2.5–15.8 with poor collaterals vs. 6.6 ml; IQR −4.1–26.5 with good collaterals; *p* = 0.62). In multiple regression analysis, after adjusting for baseline infarct volume and reperfusion status, worse baseline collaterals (higher HIR) showed a trend for increased early infarct growth (*p* = 0.06). In contrast, patients who did not reperfuse had similar volumes of median early infarct growth (38.4 ml; IQR 20.9–79.0; *p* = 0.19) compared to patients with reperfusion (26.7 ml; IQR 14.5–49.3), but showed increased late infarct growth (11.2 ml; IQR −1.1–27.2 without reperfusion vs. 1.9 ml; IQR −6.1–8.5 with reperfusion; *p* < 0.01, Figure 1B). In multiple regression analysis, after adjusting for baseline collateral status, the absence of reperfusion remained associated with increased late infarct growth (*p* = 0.04). The interaction term between collateral and reperfusion status was not a significant predictor of either early or late infarct growth.

To ensure that late infarct growth was not exaggerated by differences in imaging modality, a sensitivity analysis excluded patients who had their 24-h infarct volume assessed on CT and their late infarct volume on MRI (*n* = 5). The results of the sensitivity analysis (*n* = 61) were similar to the main results, with early infarct growth showing an association with baseline collateral status and late infarct growth showing a trend with reperfusion status (Supplementary Table 1).

## Discussion

This study assessed infarct volume at three time points, allowing infarct growth to be investigated independently within discrete early and late time windows. The results suggest that both baseline collateral blood flow and large vessel reperfusion influence infarct growth, but that their effects are best appreciated at different time points. We found that poor baseline collateral status is associated with increased infarct growth between baseline and 24-h imaging. This finding is consistent with other studies that have examined the impact of collateral status on infarct growth in this time window (10). Secondly, we found that absence of reperfusion is associated with increased infarct growth beyond 24-h. This is consistent with Federau et al. (3) who demonstrated that reperfusion status influences infarct growth over an extended time period (5 days) and that the effect of reperfusion on infarct growth is not fully appreciated shortly following endovascular therapy.

**Figure 2:**
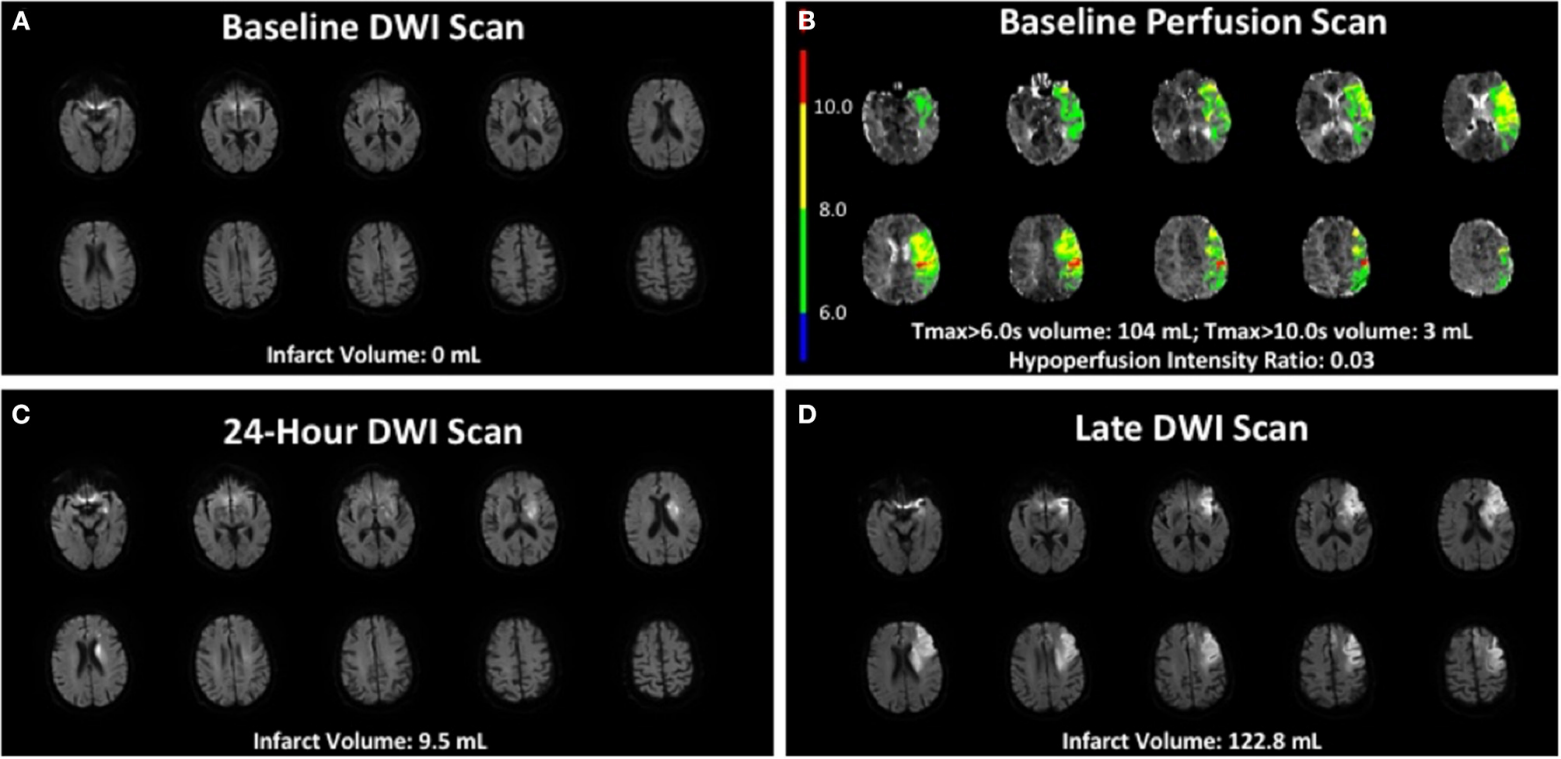
Case example of baseline DWI and perfusion scan, a 24-h DWI scan, and a late DWI scan. Seventy-eight-year-old patient with 12 h of symptoms prior to baseline MRI, with good baseline collaterals but who did not reperfuse with imaging demonstrating minimal early infarct growth and substantial late infarct growth. **(A)** Baseline MRI showed a 0 ml DWI infarct. **(B)** Baseline perfusion imaging showed good collaterals with a hypoperfusion intensity ratio of 0.03. **(C)** 24-h MRI showed a 9.5 ml DWI infarct. **(D)** Late MRI scan occurred 12 days after 24-h scan and showed a 122.8 ml DWI infarct volume.

In the multivariate analysis predicting late growth, there was a trend for HIR (*p* = 0.07) suggesting that patients with lower HIR (better baseline collaterals) had increased late infarct growth (Table 2). This may seem paradoxical as the effect is opposite to the direction in the early window in which good collaterals predict decreased growth. This finding is, however, consistent with Campbell et al. (4) who suggest that good collaterals contribute to a large mismatch between infarcted and hypoperfused tissue, limited early infarct growth, and substantial infarct growth in the late time window if the collaterals fail. This is illustrated in Figure 2 which shows the DWI and perfusion imaging of a patient with good baseline collaterals who fails to reperfuse. This patient experiences limited early infarct growth but substantial late infarct growth. We speculate that this patient's late infarct growth is the result of failure of collaterals in the absence of reperfusion. While we were unable to assess collateral failure directly as we did not have perfusion imaging with the late scan, other studies have established that collateral status declines over time, and that collateral deterioration is associated with infarct growth (4, 5).

**Table 2:** Multiple linear regression analysis for infarct growth (*n* = 66).

**Variable**	**Early infarct growth**		**Late infarct growth**	
	**Growth (ml)**	***p*-value**	**Growth (ml)**	***p*-value**
Intercept	−6.46	0.69	11.89	0.13
HIR at baseline	83.58	0.06	−29.26	0.07
Reperfusion status (non-reperfuser)	14.02	0.33	14.75	0.04
Infarct volume at baseline (ml)	1.33	0.003	-	-

*HIR, Hypoperfusion intensity ratio; Early Infarct Growth defined as growth between baseline and 24 h; Late Infarct Growth defined as growth between 24 h and 5 days*.

In contrast to patients with good baseline collaterals, the infarcts of patients with poor baseline collaterals grow quickly and reach their final volume early. As a result, these patients typically show only limited growth beyond the first 24 h.

Our results may explain the paradoxical results, reported previously, that in the DEFUSE 3 trial good baseline collateral status was not predictive of improved functional outcome despite showing reduced infarct growth at 24 h (10). Our study, conducted in the subset of the DEFUSE 3 population who underwent a late unscheduled scan, suggests that this finding might be explained by continued infarct growth beyond 24 h, particularly in patients with good baseline collaterals who failed to reperfuse (11).

There are several limitations to this study. First, this study's findings are specific to patients who met DEFUSE 3 selection criteria and therefore have relatively slow infarct progression and small ischemic core volumes at baseline and caution must be used in extrapolating these findings to a more general stroke patient population. Secondly, this study had a limited sample size because it relied upon unscheduled late window scans. This introduces a bias toward patients with worse outcomes who are more likely to undergo repeat scans, which is evidenced by the lower rate of reperfusion, higher 24-h NIHSS score, and higher death rate at day 90 in this study compared to the overall DEFUSE 3 study. However, it is unlikely that this bias influenced the results of our analyses, which were focused on the role of collateral status and reperfusion on lesion growth. Thirdly, the timing of the late scan was variable. This, however, likely did not impact the results as the timing of the late scan was not correlated with late infarct growth. Third, the imaging modality of the late scan was variable. However, a sensitivity analysis removing all cases who underwent CT at 24 h and an MRI, which typically shows larger infarcts, at the later time-point, showed similar results to the overall analysis. Another limitation of this study is that reperfusion was assessed at 24 h and we were unable to account for cases that spontaneously recanalized at a later time point. Finally, this study would have benefited from perfusion data obtained during the follow-up scans which would have allowed us to assess the evolution of collateral status over time.

In conclusion, this study demonstrates that infarct growth depends on different factors during different time windows. In the DEFUSE 3 patient population poor baseline collateral blood flow is a strong predictor of infarct growth in the first 24 h after enrollment, whereas absence of large vessel reperfusion is a strong predictor of infarct growth after 24 h. Our results highlight the need for timely reperfusion therapy, particularly in patients with poor collaterals and indicate that the 24-h timepoint is too early to assess the full impact of reperfusion therapy on infarct growth. Future studies evaluating infarct evolution should consider investigating these factors in discrete early and late time windows.

## Data Availability Statement

The raw data supporting the conclusions of this article will be made available by the authors, without undue reservation.

## Ethics Statement

Ethical approval for this study was obtained from StrokeNet Central Institutional Review Board. The patients/participants provided their written informed consent to participate in this study.

## Author Contributions

WT and ML: experimental design. WT, LP, SC, MM, SK, JH, MPM, GA, and ML: analysis/interpretation of data and manuscript writing and editing. All authors contributed to the article and approved the submitted version.

## Conflict of Interest

GA has equity in and is a consultant for iSchemaView, which produces the software used in DEFUSE 3 for postprocessing of computed tomography and magnetic resonance perfusion studies. He also holds a patent related to that software and has been a consultant for Medtronic. JH is a consultant for Medtronic and MicroVention and a member of the iSchemaView Medical and Scientific Advisory Board. MPM is a shareholder in ThrombX Medical. The remaining authors declare that the research was conducted in the absence of any commercial or financial relationships that could be construed as a potential conflict of interest.
