# Supplementary material for: Predictors of Early and Late Infarct Growth in DEFUSE 3
Source: Front Neurol. 2021 Jul 1;12:699153. doi: 10.3389/fneur.2021.699153 (PMC8283804; doi:10.3389/fneur.2021.699153)
Supplement: Supplementary file 1 [file Table_1.docx]

**Supplementary Table 1.** Sensitivity Analysis – Multiple linear regression analysis for infarct growth removing the five cases for whom 24-hour infarct volume was assessed on CT while late infarct volume was assessed on MRI (n = 61).

|  | **Early Infarct Growth** | | |  | | | **Late Infarct Growth** | | |
| --- | --- | --- | --- | --- | --- | --- | --- | --- | --- |
| **Variable** | **Growth (mL)** | | **p Value** | |  | | **Growth (mL)** | **p Value** | |
| Intercept | -10.12 | 0.57 | | | |  | 12.93 | | 0.13 |
| HIR at baseline | 90.69 | 0.05 | | | |  | -29.69 | | 0.09 |
| Reperfusion status  (Non-Reperfuser) | 17.95 | 0.25 | | | |  | 13.14 | | 0.08 |
| Infarct volume at baseline (mL) | 1.29 | <0.01 | | | |  | - | | - |

Note: HIR, Hypoperfusion intensity ratio; Early Infarct Growth defined as growth between baseline and 24 hours; Late Infarct Growth defined as growth between 24 hours and 5 days.
